# *Streptococcus constellatus* Causing Septic Thrombophlebitis of the Right Ovarian Vein with Extension into the Inferior Vena Cava

**DOI:** 10.1155/2015/495898

**Published:** 2015-06-16

**Authors:** Abdallah Haidar, Amy Haddad, Amir Naqvi, Ngozi U. Onyesoh, Rushdah Malik, Michael Williams

**Affiliations:** ^1^Department of Internal Medicine, Providence Hospital and Medical Center, 16001 W. Nile Mile Road, Southfield, MI 48075, USA; ^2^Department of Infectious Disease, Providence Hospital and Medical Center, 16001 W. Nile Mile Road, Southfield, MI 48075, USA

## Abstract

*Introduction*. *Streptococcus constellatus* collectively with *Streptococcus anginosus* and *Streptococcus intermedius* constitute the *Streptococcus anginosus* (formerly *Streptococcus milleri*) group. Though they are commonly associated with abscesses, bacteremia with subsequent septic thrombophlebitis is extremely rare, and resulting mortality is infrequent. *Case Presentation*. We report a case of a previously healthy 60-year-old African American female who presented with *Streptococcus constellatus* bacteremia associated with septic thrombophlebitis to the right ovarian vein extending into the inferior vena cava. She was urgently treated with antibiotics and anticoagulation. *Conclusion*. Septic thrombophlebitis has a clinical presentation that is often misleading. Therefore, a high clinical index of suspicion and the use of appropriate imaging modalities (computed tomography) are essential in recognizing and confirming this diagnosis. Prompt treatment is warranted. Surgical thrombectomies have been successfully replaced by a combination of antibiotics and anticoagulation therapy.

## 1. Case Report

**Figure 1:**
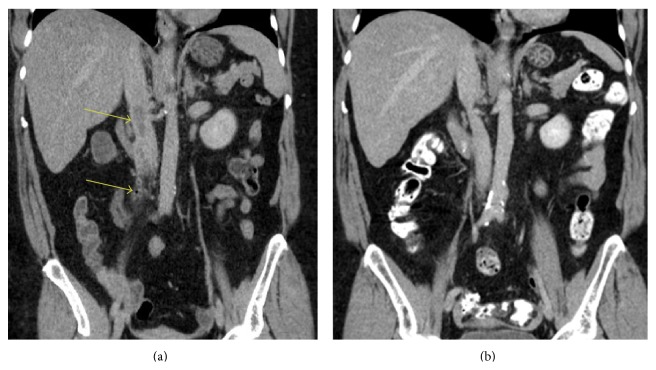
(a) Septic deep vein thrombosis of the right ovarian vein with extension into the inferior vena cava. Dilation of the right ovarian vein, with a filling defect and air bubble, is seen in the proximal right ovarian vein, surrounded by fat stranding (lower arrow). This filling defect extends into the IVC at the level of the renal veins (upper arrow). These findings are consistent with septic deep thrombophlebitis of the proximal right ovarian vein with extension into the IVC. (b) 2.5 months later, there has been a complete resolution of septic thrombophlebitis of the right ovarian vein.

A 60-year-old African American female with no known prior medical history presented to the emergency department with complaint of intermittent fever, nausea, and vomiting for 2-week duration, in addition to a dry cough for five days. Also notable in her history was a recent travel to Dallas and Las Vegas from Michigan 1 week prior to presentation. Initially, she had a septic presentation with a blood pressure of 82/45 mm Hg, fever of 101 degrees Fahrenheit, leukopenia with a white blood cell count (WBC) of 3.5 k/mcl, a respiratory rate of 22 breaths per minute, and a rapid heart rate of 150 bpm. After hemodynamic stabilization with intravenous fluids, a computed tomography (CT) of abdomen and pelvis with contrast was performed and it demonstrated a septic deep venous thrombosis of the ovarian vein with extension into the inferior vena cava (IVC), seen in Figure 1(a). Anticoagulation with intravenous heparin and broad-spectrum antibiotics (ceftriaxone, gentamicin, and metronidazole) were initiated. Blood cultures from two bottles collected prior to antibiotic administration grew* Streptococcus constellatus*, and the initial antibiotics were changed to ampicillin-sulbactam. The pelvic and transvaginal ultrasound tests were unremarkable. Urine and cervical cultures were negative. Chest X-ray findings included right upper lobe infiltrates concerning pneumonia. CT-thorax demonstrated a 1.1-centimeter nodule in the right lung and airspace disease in the right middle lobe suspicious for pneumonia superimposed on emphysema, but it was negative for acute pulmonary embolism. Echocardiogram was normal with no vegetation visualized. The patient remained free of fever for more than 24 hours prior to discharge. She was also bridged after 24 hours to warfarin for continued anticoagulation therapy and remained hemodynamically stable and asymptomatic. She was discharged home in stable condition after a 5-day hospitalization; she was continued on oral antibiotics (amoxicillin and clindamycin) for 10 days, as well as oral anticoagulation (warfarin) for 3 months. The patient was followed up as an outpatient and was clinically doing well with no complaints and had remained persistently afebrile; her international normalized ratio (INR) remained therapeutic. A repeated CT two and a half months later showed a complete resolution of the septic thrombophlebitis of the right ovarian vein and IVC, Figure 1(b). Also there has been a complete resolution of the pulmonary nodule which may suggest septic emboli to the lung versus upper respiratory infection at presentation.

## 2. Background

*Streptococcus constellatus* is a species of group C nonhemolytic viridans streptococci. It is gram-positive, nonspore forming, nonmotile, catalase-negative cocci.* Streptococcus anginosus*,* Streptococcus intermedius*, and* Streptococcus constellatus* together make up the anginosus group (formerly milleri group). This group was first found in dental abscesses in 1956 [1]. It is usually considered part of the normal flora of the respiratory, gastrointestinal, and genitourinary tract. However, it can cause systemic purulent infections. Septic thrombophlebitis can be one of its rare clinical presentations, as was the case with our patient. Due to their nonspecific symptoms, including fever and lack of physical findings, early diagnosis of septic thrombophlebitis requires high clinical suspicion as well as the use of appropriate imaging techniques [2]. We present a rare case of septic pelvic thrombophlebitis caused by* Streptococcus constellatus* in an immunocompetent 60-year-old female.

## 3. Discussion

Septic pelvic thrombophlebitis is an uncommon entity, with approximately 4 cases occurring per year as seen in one study [13]. Furthermore, cases of septic pelvic thrombophlebitis caused by* S. constellatus* are extremely rare. When it is seen,* S. constellatus* is more common among intravenous drug abusers [5, 14]. Up to our knowledge, including our case, there have been eleven cases of septic thrombophlebitis reported in the literature where* S. constellatus* was involved either alone or among other bacteria; see Table 1. The majority of cases were located in the head and neck (7 out of 11); of those, cavernous sinus thrombophlebitis was the most commonly reported location. The remaining four cases were located in the intra-abdominal and pelvic area. Besides this case, three other cases were described: septic iliofemoral thrombophlebitis in an IV drug abuser [5], septic thrombosis of the portal vein and its branches (pylephlebitis) as a complication of diverticulitis [3], and a case of appendicitis that was complicated by portal pylephlebitis [4]. Predisposing factors for those eleven cases were variable.

Other bacteria, such as* Staphylococcus aureus*, are more commonly associated with septic thrombophlebitis. In a small study,* Staphylococcus aureus* was found in 4 out of 7 patients who had septic thrombophlebitis, while other bacteria isolated included coagulase-negative* Staphylococcus* species,* Proteus mirabilis*, and* Propionibacterium* species [14].* Streptococcus constellatus* has been associated with other infectious presentations including septic shock after tooth extraction [15], mitral and aortic valve endocarditis [16], and subdural empyema [17].

*S. constellatus* had previously been reported more frequently as part of the milleri group (currently anginosus group). In a UK study [18], 151 strains of milleri group were collected from multiple laboratories. There have been 33 total cases of* S. constellatus* previously identified, and no predominant body site was found to be associated with this organism. Three cases were associated with genitourinary clinical source and four were isolated from other abdominal and pelvic sites. On the other hand, the total number of* S. anginosus* identified was 89, with predominant source being the genitourinary and gastrointestinal tracts, 40 and 19, respectively. The infections of the central nervous system (CNS) were most commonly associated with* S. intermedius* strains; 18 out of 29 total strains were identified.

*Streptococcus constellatus* has often been associated with abscesses. Clarridge III et al. [19] reported 118 patients with confirmed isolates of the* Streptococcus milleri* group, of which 54 patients (46%) had a total of 56 isolates of* S. constellatus*. In 41 (73%) of the 56 isolates obtained,* S. constellatus* was involved in abscess formation.

Bacteremia from the* Streptococcus anginosus* group is infrequent. Knowledge of the different clinical presentations may aid in determining the source of the infection. In our case, however, the exact etiology and source of septic thrombophlebitis of the right ovarian vein remained unclear. Dehydration and recent travel history could have been the predisposing factors contributing to deep venous thrombosis (DVT) in our case. In general, septic pelvic thrombophlebitis is seen more often among younger females in their reproductive age. Its risk factors include underlying malignancy, pelvic infection, uterine fibroids, and severe dehydration. Other factors include cesarean section, pregnancy, hormonal stimulation, induced abortion, and a postpartum state in nonmenopausal females, none of which was present in our patient. Ovarian vein thrombosis and septic pelvic thrombophlebitis have predominantly been described in young women who are postpartum or after surgery compared to our case in a postmenopausal woman. The clinical presentation related to these entities has been reported to include ipsilateral abdominal pain, fever, flank or back pain, nausea or vomiting, and leukocytosis on laboratory findings [20–22].

Patients with septic thrombophlebitis are at risk of pulmonary embolism, especially when there is involvement of the IVC. Therefore, immediate treatment is important. Previously, the treatment of septic thrombophlebitis involved surgery [23]. However, anticoagulation and antibiotic therapies have since been proven to be efficacious in the treatment of septic thrombophlebitis, with no recurrence after treatment [14]. Broad-spectrum antibiotics are recommended as part of the initial treatment course. Blood cultures are frequently negative, making it difficult to define commonly responsible organisms. The most commonly involved pathogens are* E. coli*,* Bacteroides* spp., other coliforms, or streptococci.* S. aureus* is less common without the appropriate predisposing risk factors. Gram-negative pathogens are seen more often in patients with intra-abdominal pathology [24]. 14% of the cases have had multiple causative agents isolated. One case series involved 46 patients with septic pelvic thrombophlebitis, including seven patients with significant involvement of the ovarian vein. In this report, the treatment course associated chloramphenicol and penicillin for antimicrobial coverage and heparin for anticoagulation. Of the 46 patients described, 42 patients were subsequently without fever within 7 days (mean: 2.5 days) [25]. The duration of intravenous therapy can be as short as 7 days if there are no other complications, followed by a course of oral antibiotics. There is a lack of definitive studies or recommendations to guide the duration of anticoagulation therapy for this entity, but one source recommends the use of enoxaparin at a dose of 1 mg/kg daily or warfarin with a goal INR of 2.5 for 3–6-month duration if the thrombosis involves the ovarian vein. This can be followed by a repeat CT scan to determine resolution of the clot after 3 months, at which point anticoagulation can be discontinued if CT is negative or continued for an additional 3 months if CT is positive [2]. Surgical thrombectomies are typically reserved for cases that fail conservative management.

## 4. Conclusion

Septic pelvic thrombophlebitis, although rare, can be associated with high morbidity. Knowing the underlying etiologies and possible masquerading presentation will help in its recognition. Instituting diagnostic techniques such as CT will also aid in its diagnosis. Rapid institution of therapy including the appropriate antibiotic in combination with anticoagulation is recommended. Further studies are needed on the duration of anticoagulation therapy. Surgery is no longer first line of therapy. Underlying etiologies should be investigated. Overall, prognosis is favorable if detected and treated early.

## Figures and Tables

**Table 1:** Cases reported in the literature of septic thrombophlebitis with confirmed *Streptococcus constellatus* involvement.

Case number	Age	Sex (M/F)	Anatomic location/clinical diagnosis	Predisposing factors	Source
1	60	F	Ovarian vein with an extension into inferior vena cava	Possibly travel and dehydration	This case report

2	63	M	Portal vein thrombosis	Complication of perforated diverticulitis	Van De Wauwer and Irvin, 2005 [3]
3	13	M	Portal vein thrombosis	Complication of appendicitis	Sakalkale and Reeve, 2006 [4]
4	24	M	Iliofemoral deep vein thrombosis	Intravenous drug abuse	Sulaiman et al., 2011 [5]
5	56	F	Cavernous sinus thrombosis complicated by narrowing of the internal carotid artery, subarachnoid abscess, and multiple pulmonary septic emboli	No contributory medical history	Hoshino et al., 2007 [6]
6	39	M	Cavernous sinus thrombosis	Chronic alcohol consumption	Chang et al., 2003 [7]
7	54	F	Facial abscess, cavernous sinus thrombosis (CST), bilateral internal jugular thrombosis, and multiple lung abscesses	Mandibular dental infection, immunosuppressed	Jones and Arnold, 2009 [8]
8	45	M	Cavernous sinus thrombosis	Severe periodontitis	Imholz et al., 2014 [9]
9	51	F	Cavernous sinus, maxillary vein, and multiple pulmonary nodular lesions (*a Lemierre syndrome variant*)	Acute otolaryngologic infection	Yamaguchi et al., 2010 [10]
10	56	M	Cavernous sinus thrombosis	Unknown origin, possibly prior endoscopic retrograde biliary drainage	Hung et al., 2014 [11]
11	52	F	Cavernous sinus thrombosis and meningitis	Chronic sinusitis and complication of osteoporosis, including orthopedic surgery	Chung et al., 2014 [12]
